# Exogenous pyruvate protects IMS32 Schwann cells from impairing the maturation process under hyperglycemia

**DOI:** 10.1016/j.jphyss.2026.100104

**Published:** 2026-10-03

**Authors:** Hideji Yako, Sana Hattori, Sayaka Iwamoto, Tatsuya Ooki, Waraku Nagatani, Yuika Kaburaki, Yuki Miyamoto, Kazunori Sango, Junji Yamauchi

**Affiliations:** a Laboratory of Molecular Neuroscience and Neurology, Tokyo University of Pharmacy and Life Sciences, Horinouchi, Hachioji, Tokyo, 192-0392, Japan; b Laboratory for Drug Target Discovery, Tokyo University of Pharmacy and Life Sciences, Horinouchi, Hachioji, Tokyo, 192-0392, Japan; c Department of Pharmacology, National Research Institute for Child Health and Development, Okura, Setagaya, Tokyo, 157-8535, Japan; d Brain Metabolic Regulation Group Frontier Laboratory, Department of Psychiatry and Behavioral Sciences, Tokyo Metropolitan Institute of Medical Science, Tokyo, 156-8506, Japan; e Department of Biological Science, Tokyo College of Biotechnology, Kitakojiya, Oota, Tokyo, 144-0032, Japan

**Keywords:** Exogenous pyruvate, Hyperglycemia, Oxidative stress, Schwann cells, Maturation

## Abstract

Peripheral neuropathy is a chronic common complication of diabetes mellitus. Continuous hyperglycemia induces metabolic disturbances and elicits oxidative stress. However, the impact of hyperglycemia on the maturation of Schwann cells is not fully understood. Therefore, we examined whether hyperglycemia affects the maturation process of IMS32 Schwann cells treated with forskolin, a Schwann cell maturation enhancer, using promoter assays and immunoblotting of Schwann cell markers such as MBP, CNPase, and GFAP. IMS32 cells exposed to high-glucose conditions showed decreased expression of these markers. Exogenous pyruvate plays a crucial role in maintaining glucose metabolism and reducing oxidative stress under hyperglycemic conditions. These decreases were prevented by pyruvate supplementation. Pyruvate treatment also prevented IMS32 cells from producing reactive oxygen species and from impairing mitochondrial membrane potential. These results indicate that hyperglycemia impairs Schwann cell maturation accompanied by mitochondrial dysfunction and oxidative stress, contributing to the development and progression of diabetic peripheral neuropathy.

## Highlights

• Hyperglycemia impaired the maturation of IMS32 Schwann cells.
• Hyperglycemia enhanced oxidative stress during Schwann cell maturation.
• Exogenous pyruvate recovered impaired maturation by attenuating oxidative stress.
• Impaired Schwann cell maturation is one of the pathogenic mechanisms of DSPN.

## Graphical Abstract

**Figure:**
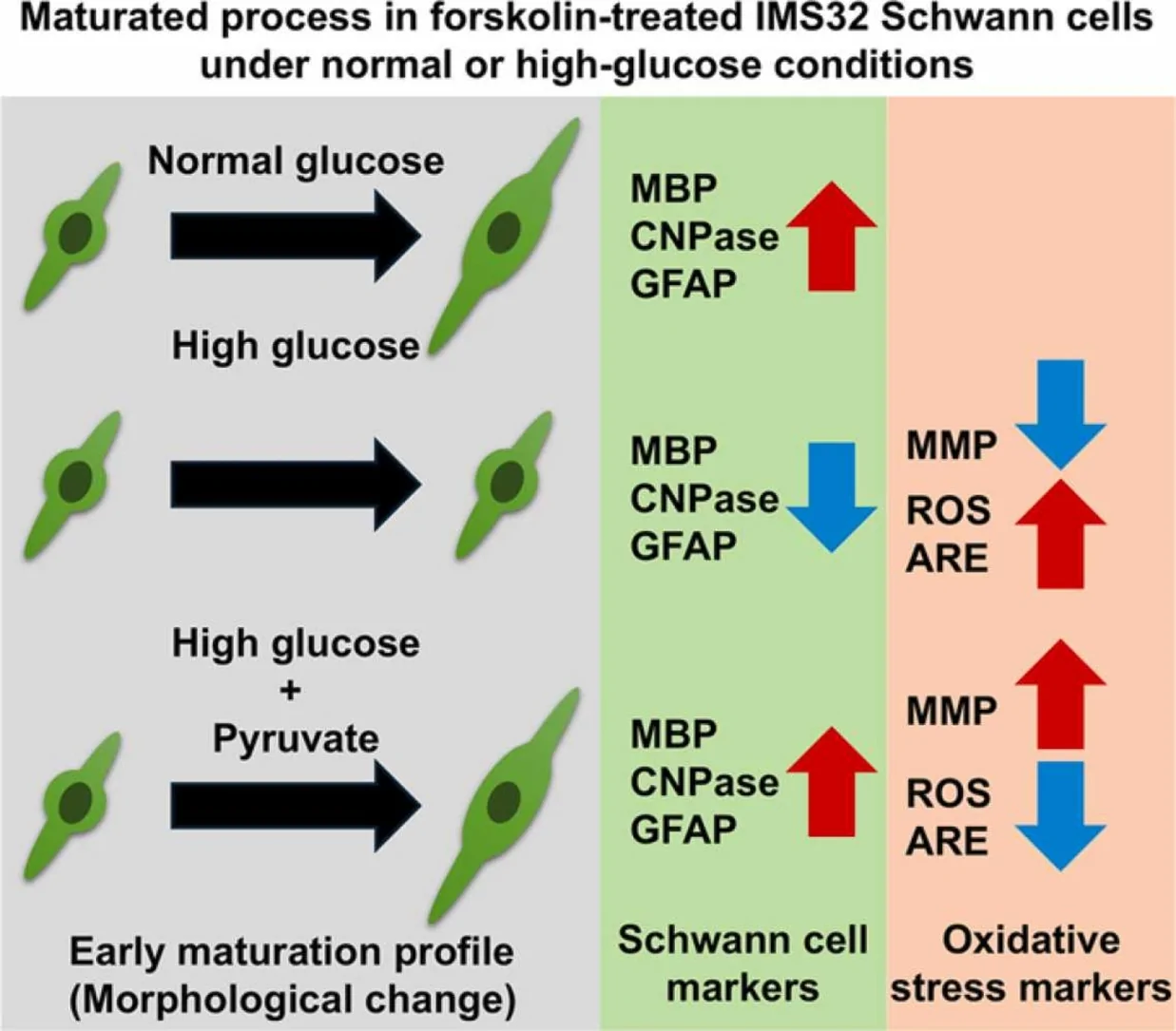

## Introduction

Peripheral neuropathy is one of the major chronic complications of diabetes mellitus. The development and progression of diabetic sensorimotor polyneuropathy (DSPN) are suggested to result from continuous hyperglycemia-induced impairment of the microvascular supply and metabolism in the peripheral nervous system (PNS). These abnormalities lead to mitochondrial dysfunction and oxidative stress, including excess reactive oxygen species (ROS) production in neurons and Schwann cells [1,2]. However, no effective pathogenesis-based therapy has yet been developed other than normalizing blood glucose levels. Therefore, investigating the pathogenesis of DSPN is essential for its prevention and treatment.

Schwann cells, the glial cells of the PNS, maintain neuronal survival and function through metabolic and neurotrophic support. Schwann cells originate from neural crest cells and differentiate into myelinating and non-myelinating Schwann cells through tightly regulated maturation processes [3,4]. Although many factors are involved in these processes [5,6], the effects of hyperglycemia remain incompletely understood. Hyperglycemia enhances dedifferentiation or abnormal myelination in sciatic nerves, primary cultured Schwann cells, and the IMS32 Schwann cell line [[7], [8], [9]]. However, the molecular mechanism during these processes remains unclear.

IMS32 cells are often used in DSPN studies [7,[10], [11], [12], [13], [14], [15], [16], [17], [18], [19], [20], [21], [22], [23], [24]] because they express and upregulate aldose reductase, a rate-limiting enzyme in the polyol pathway, under hyperglycemic conditions [[10], [11], [12],^17^,^25^,^26^]. These changes enhance glucose flux through the polyol pathway and oxidative stress, which is a major pathogenic mechanism and therapeutic target in DSPN [25,[27], [28], [29], [30], [31], [32], [33], [34], [35]]. However, when treated with forskolin, an activator of Schwann cell maturation, IMS32 cells upregulate Schwann cell markers such as 2′,3′-cyclic nucleotide 3′-phosphodiesterase (CNPase) and glial fibrillary acidic protein (GFAP) [36]. These findings suggest that IMS32 cells retain characteristics of immature Schwann cells, even though hyperglycemia contributes to a dedifferentiated phenotype [[7], [8], [9]]. Although our previous studies indicated that hyperglycemia induced oxidative stress and cell death in IMS32 cells [13,14], the effects of hyperglycemia on Schwann cell maturation and the molecular mechanisms underlying this process remain poorly understood. Therefore, we investigated the effect of hyperglycemia on Schwann cell maturation in IMS32 cells treated with forskolin. Our previous findings indicated that exogenous pyruvate attenuated oxidative stress and restored cell viability in IMS32 cells under high-glucose conditions [14,16]. In addition, pyruvate treatment ameliorated hyperglycemia-induced lesions in ND7/23 cells, a dorsal root ganglion neuron cell line, and partially improved sensory dysfunction in streptozotocin-induced diabetic mice [37]. However, whether pyruvate protects Schwann cell maturation under hyperglycemia remains unknown. Therefore, we assessed whether exogenous pyruvate could ameliorate impaired maturation under hyperglycemic conditions. Our findings provide insight into the mechanisms underlying Schwann cell maturation under high-glucose conditions and may contribute to the development of pathogenesis-based therapies for DSPN.

## Materials and methods

### Key materials

The key materials used in this study are presented in Table 1.

**Table 1:** Key materials and reagents in this manuscript.

Reagents or materials	Companies or sources	Cat. Nos.	Lot. Nos.	Experimental conditions
Antibodies				
Anti Myelin basic protein (MBP) rabbit polyclonal antibody	MBL	PD004	014	Immunoblotting (IB), 1:5000
Anti mouse 2′, 3′-cyclic nucleotide 3′-phosphodiesterase (CNPase) mouse monoclonal antibody	Santa Cruz Biotechnology	sc-166019	L2216	IB, 1:500
Anti human glial fibrillary acidic protein (GFAP) rabbit polyclonal antibody	Proteintech	16825–1-AP	00168568	IB, 1:500
Anti β Actin mouse monoclonal antibody	MBL	M177–3	007	IB, 1:10,000
Anti rabbit IgG (goat) pre-absorbed HRP-conjugate	Nacalai Tesque	21858–24	M3H6046	IB, 1:10,000
Anti mouse IgG (goat) pre-absorbed HRP-conjugate	Nacalai Tesque	21860–61	L4B5968	IB, 1:10,000
Plasmids				
pRP[Exp]-MBPp-EGFP (mouse MBP promoter and EGFP in tandem)	VectorBuilder	pDNA(VB231221–1509zhe)	VB231221–1509zhe-1	6 μg of DNA per 3.5 cm dish
pRP[Exp]-Cnp-EGFP (mouse CNPase promoter and EGFP in tandem)	VectorBuilder	pDNA(VB230819–1002rec)	VB230819–1002rec-1	6 μg of DNA per 3.5 cm dish
pRP[Exp]-GFAP(long)-EGFP (human GFAP long version promoter and EGFP in tandem)	VectorBuilder	pDNA(VB230819–1003mgs)	VB230819–1003mgs-1	6 μg of DNA per 3.5 cm dish
SV40PA-ARE pcDNA3.1 (+)-C-eGFP (human antioxidative stress element and EGFP in tandem)	GenScript	-	J573E835G0–2	6 μg of DNA per 3.5 cm dish

### Cell culture and maturation

Spontaneously immortalized adult mouse Schwann cell line IMS32 cells (Cosmo Bio, Tokyo, Japan) were maintained in Dulbecco’s modified Eagle medium (low glucose) containing 5.6 mM glucose and 1 mM pyruvate (DMEM; Nacalai Tesque, Kyoto, Japan), supplemented with 5% heat-inactivated fetal bovine serum (FBS; Thermo Fisher Sciencetific, Waltham, MA, USA) and an antibiotic–antimycotic mixed solution (100 units/mL of penicillin and 100 μg/mL of streptomycin; Nacalai Tesque Inc.) as the basal medium in 5% CO_2_ at 37°C. IMS32 cells were seeded at approximately 80% confluency. To enhance maturation, IMS32 cells at approximately 80% confluency were exposed to growth medium containing 5 μM forskolin (Nacalai Tesque Inc.) in 5% CO_2_ at 37°C, according to the previous study [36]. Morphological analyses were performed at 0 (Day 0), 1, 3, 5, 24 (Day 1), and 48 (Day 2) h after forskolin treatment. Promoter assays were performed at Day 0, Day 1, and Day 2 h after treatment, and Western blot analyses were performed at Day 2. To examine the efficacy of pyruvate, sodium pyruvate (Nacalai Tesque) was added to achieve final pyruvate concentrations of 5 and 10 mM.

### Cell morphological analysis

Morphological images of IMS32 cells were obtained using a smartphone-based i-NTER LENS system (Micronet Inc., Saitama, Japan) and i-NTER SHOT (ver.2, Micronet Inc.). Process length was assessed using ImageJ software (ver. Java 8, https://imagej.nih.gov/ [accessed on 15 December 2024]). The longest process of IMS32 cells was measured in 10 randomly selected fields. The same dataset was used to determine both the distribution of single-cell process lengths (time-course analysis) and the mean process length for statistical analysis. Mature IMS32 cells were defined as having a process length > 30 μm and the percentage of mature cells was calculated from 10 randomly selected fields.

### Plasmid transfection and differentiation marker promoter assay

IMS32 cells at approximately 80% confluency were transfected with 6 μg of plasmids (Table 1) using 2.5 μl of HilyMAX (DOJINDO, Japan) in accordance with the manufacturer’s instructions. The medium was replaced 4 h after transfection with DMEM containing 5.6, 25, 50 or 100 mM glucose (high-glucose) and 100 mM mannitol, supplemented with 1, 5 or 10 mM sodium pyruvate or 150, 300 or 600 μg/mL N-acetyl cysteine (NAC; Nacalai Tesque Inc.), 5% heat-inactivated FBS, an antibiotic–antimycotic mixed solution, and 5 μM forskolin, and the cells were maintained for 1 or 2 days. The proportion of EGFP-positive cells 2 days after transfection with pEGFP-C1 (Takara Bio) reached approximately 80% (Figure S1). No significant differences in the proportion and cell viability of EGFP-positive cells were observed among the experimental conditions (Figure S2). EGFP and phase-contrast images were obtained using a microscope (BX-700 or BZ-X1000, Keyence, Osaka, Japan). Under these conditions, the estimated percentage of attached cells incorporating trypan blue was < 5% in each experiment. IMS32 cells were immersed in 0.05% trypan blue solution (Nacalai Tesque Inc.) for 10 min. After washing with PBS, trypan blue- positive and negative cells were observed under the microscope.

### ARE promoter assay

To evaluate Nrf2 transcriptional activity, IMS32 cells were transfected with an ARE-driven EGFP reporter plasmid (Table 1) under the same conditions as in the section of *Plasmid transfection and differentiation marker promoter assay*. The proportion of EGFP-positive cells was quantified using the same procedure as described for the Schwann cell marker promoter assays.

### Immunoblotting

IMS32 cells were dissolved in a buffer containing 50 mM Tris-HCl, pH 7.4, 150 mM NaCl, 5 mM MgCl2, 1 mM dithiothreitol, 1 mM phenylmethane sulfonylfluoride, 1 μg/mL leupeptin, 10 mM EDTA, 1 mM Na3VO4, 10 mM NaF, and 0.5% NP-40 at 4°C. Lysates were centrifuged at 10,000 rpm for 10 min at 4°C, and the supernatants were collected. Samples were denatured in sample buffer (Fujifilm Wako Chemical, Japan). After determining protein concentration using the Bradford assay, samples were applied onto homemade polyacrylamide gels and then transferred to polyvinylidene fluoride membranes (EMD Millipore, Burlington, MA, USA). Target proteins were visualized using the antibodies described in Table 1. Bands of interest were detected using Chemi-lumi One Ultra or Chemi-lumi One Super (Nacalai Tesque Inc.) in accordance with the manufacturer’s instructions and captured using a chemiluminescence imaging system (EZ-Capture II; ATTO, Tokyo, Japan). Band intensities were measured using ImageJ software. The relative intensity of each protein was calculated as the intensity of each protein normalized by the intensity of Actin.

### Measurement of reactive oxygen species and mitochondrial membrane potential

ROS production and mitochondrial membrane potential in IMS32 cells under the conditions described above were assessed using the ROS Assay Kit (Photo-oxidation Resistant DCFH-DA; DOJINDO) and the MT-1 MitoMP Detection Kit (DOJINDO), respectively, in accordance with the manufacturer’s instructions. Briefly, after inducing maturation under normal or high-glucose conditions supplemented with pyruvate for 2 days, cells were incubated with 10 μM DCFH-DA working solution or MT-1 working solution for 30 min in 5% CO_2_ at 37°C, respectively. After incubation, cells were washed with PBS. Images were obtained using a fluorescence microscope (BZ-X700, Keyence) under identical exposure setting between experimental groups, and fluorescence intensities were quantified using ImageJ software. Individual cells were manually selected using the ROI Manager in ImageJ and background-subtracted fluorescence intensities were measured. A total of 50 cells for ROS analysis or 80 cells for mitochondrial membrane potential analysis per condition were assessed.

### Cell viability assay

Cell viability of the treated IMS32 cells described above was confirmed using Aqueous One solution (Promega, Madison, WI, USA) in accordance with the manufacturer’s instructions. Briefly, Cultured IMS32 cells were added with 10 μl of Aqueous One solution and incubated for 2 h in 5% CO_2_ at 37°C. Absorbances at 492 nm were assessed using a microplate reader (MPR-A100, AS ONE corporation, Osaka, Japan).

### Statistical analysis

All data are presented as the mean ± standard deviation. Homogeneity of variance was assessed using Fisher's F-test prior to parametric analyses. Student's *t*-test was applied when equal variances were confirmed, whereas Welch's *t*-test was used when variances were unequal in Excel (ver. 2024, Microsoft, Redmond, WA, USA). For comparisons among multiple groups, one-way analysis of variance followed by Tukey’s honest significant difference test or Dunnett's test was performed using the StatPlus add-in for Excel. Tukey’s HSD test was used for all pairwise multiple comparisons, whereas Dunnett’s test was used when multiple groups were compared with a single control group. A *p*-value < 0.05 was considered statistically significant. Given the small sample size typical of biological experiments, data distributions were visually inspected before statistical analysis.

## Results

### Hyperglycemia impaired the maturation process in forskolin-treated IMS32 Schwann cells

We previously reported that forskolin treatment increases Schwann cell marker expression in IMS32 cells [36]; therefore, we investigated forskolin-enhanced morphological changes during IMS32 cell maturation. In the absence of forskolin, IMS32 cells exhibited a spindle-shaped morphology, whereas elongated shapes were observed in its presence in a time-dependent manner (Figure S3). A proportion of IMS32 cells showed processes shorter than 30 μm at 0 h after forskolin treatment. Process elongation progressively occurred during maturation (Figure S3D). Based on these findings, cells with processes longer than 30 μm were operationally defined as mature Schwann cells. The proportion of mature IMS32 cells increased from approximately 30–80%, reaching a peak 5 h after forskolin treatment (Figure S3). We then examined whether high-glucose conditions affected cell morphology and maturation. Hyperglycemia reduced the number of mature IMS32cells and shortened their process length (Figure S4). These findings suggest that hyperglycemia impairs the maturation of IMS32 cells.

We have also reported that forskolin-treated IMS32 cells show increased expression of Schwann cell markers such as 2′,3′-cyclic nucleotide 3′-phosphodiesterase (CNPase) and glial fibrillary acidic protein (GFAP), as assessed by promoter activity assays and immunoblot analysis [36]. To further characterize the maturation status of these cells, we employed a promoter construct for myelin basic protein (MBP), a marker of myelinating Schwann cells. The proportion of EGFP-positive IMS32 cells driven by the MBP promoter increased in a time-dependent manner under forskolin treatment (Figure S5). These positive cells were comparable to those observed using CNPase and GFAP promoter constructs (Fig. 1).

**Fig. 1:**
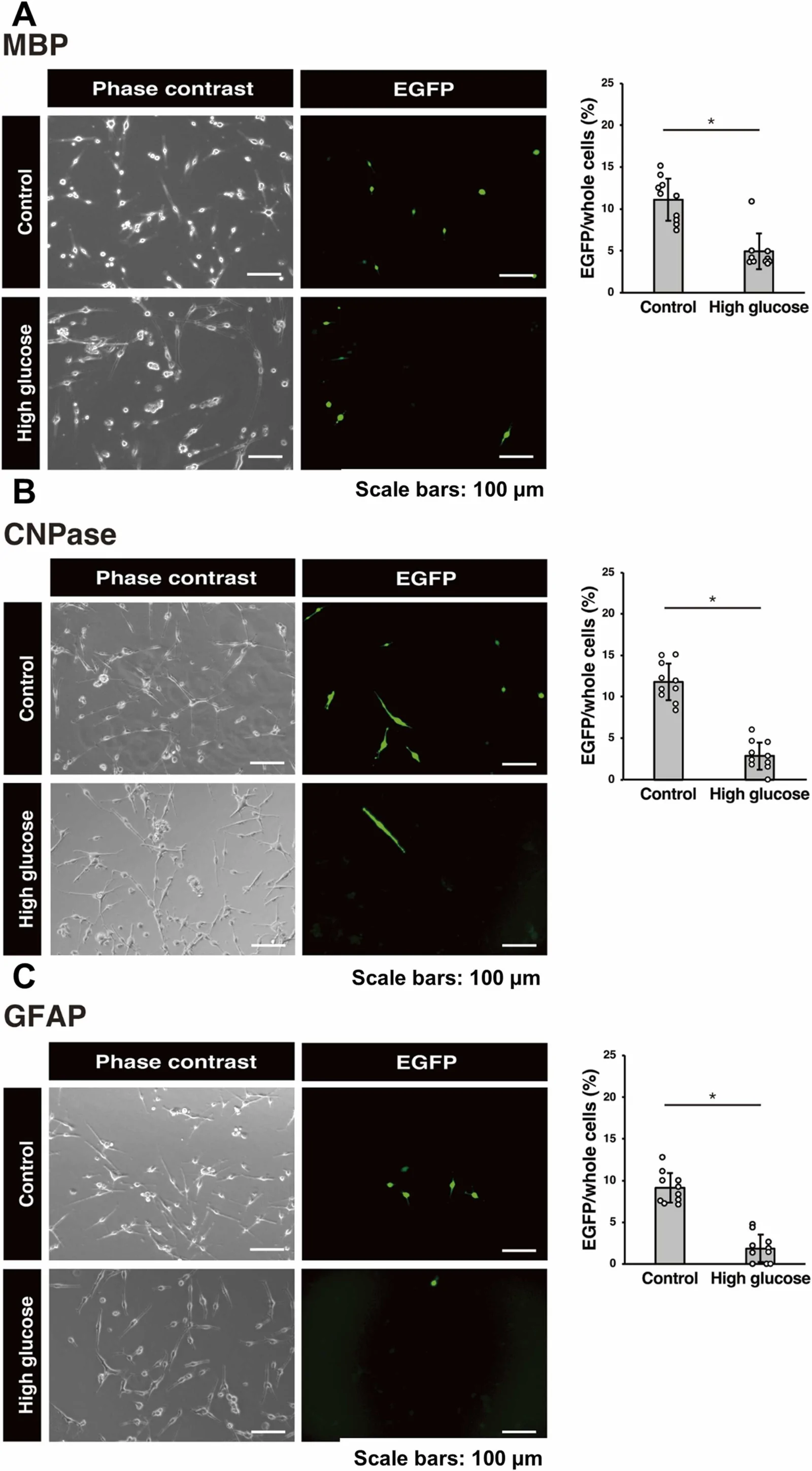
Hyperglycemia reduced the number of EGFP-positive cells driven by MBP, CNPase, or GFAP promoters in IMS32 cells treated with forskolin. Representative phase-contrast and EGFP images of IMS32 cells exposed to normal or high-glucose conditions are shown. EGFP-positive cells driven by MBP (A), CNPase (B), or GFAP (C) promoters are depicted. The bar graphs show the ratio of EGFP-positive cells under normal and high-glucose conditions. Values represent the mean ± SD, with individual values depicted as circles (n = 10 fields). *p < 0.05.

To assess the effect of hyperglycemic conditions on Schwann cell maturation, IMS32 cells were transfected with EGFP-expressing plasmids driven by the MBP, CNPase, or GFAP promoters under hyperglycemic conditions. Under normal glucose conditions, approximately 10% of IMS32 cells exhibited EGFP expression for each promoter. In contrast, under high-glucose conditions, the proportion of EGFP-positive cells driven by each promoter was significantly reduced to approximately 3% **(**Fig. 1). We also assessed the effects of glucose levels on Schwann cell maturation. The decreased EGFP-positive cells were observed at 25 mM glucose and became more pronounced at 50 and 100 mM glucose (Figure S6). We also confirmed the cell viability of IMS32 cells under 5.6, 25, 50, or 100 mM glucose, indicating that hyperglycemia did not affect the viability (Figure S2). There were mostly reductions in EGFP-positive cell driven by each promoter under 100 mM glucose; therefore, we selected 100 mM glucose conditions.

To confirm the promoter assay results, we also examined changes in MBP, CNPase, and GFAP protein levels using immunoblotting, which showed that the expression of these proteins was significantly reduced under hyperglycemic conditions compared with normal glucose conditions (Fig. 2). To investigate the impact of osmotic stress on maturation, IMS32 cells were also exposed to high-mannitol conditions. No significant differences were observed between normal glucose conditions and high-mannitol conditions in the ratio of EGFP-expressing IMS32 cells driven by the GFAP and MBP promoters **(**Figures S7 and S8). These results indicate that hyperglycemia, but not osmotic stress, impairs the maturation of IMS32 cells treated with forskolin.

**Fig. 2:**
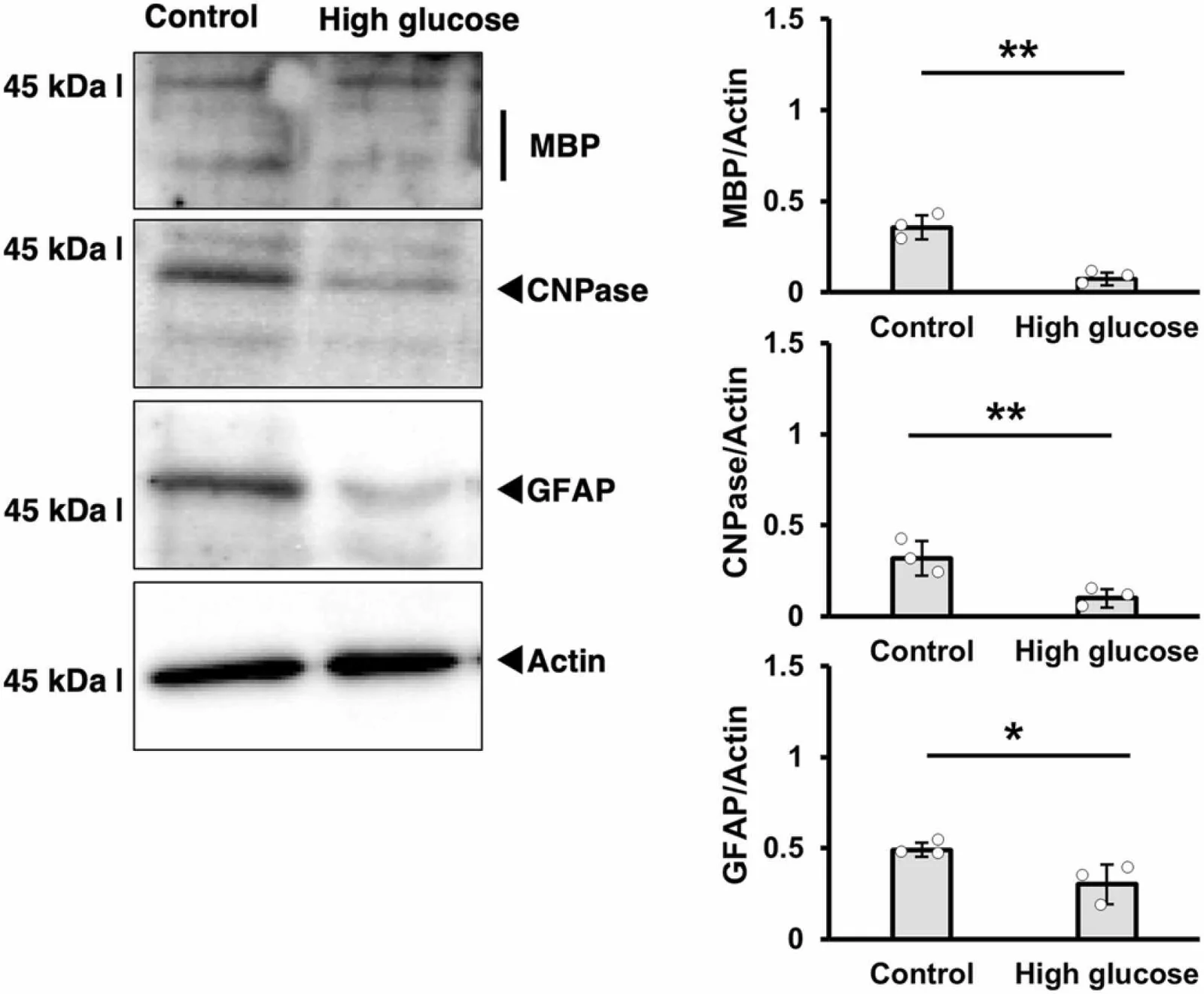
Hyperglycemia decreased Schwann cell marker expression in IMS32 cells treated with forskolin. Representative band images of MBP, CNPase, GFAP, and Actin of IMS32 cells exposed to normal or high-glucose conditions are shown. The bar graphs show the ratio of the intensity of each protein normalized by the intensity of Actin. Actual blots for MBP, CNPase, GFAP, and Actin are shown in Fig. S12. Values represent the mean ± SD, with individual values depicted as circles (n = 3). *p < 0.05, **: *p* < 0.01.

### Oxidative stress was elevated under hyperglycemia during maturation

Oxidative stress, including ROS production and mitochondrial dysfunction accompanied by hyperglycemia, is one of the pathogenic mechanisms in diabetic sensorimotor polyneuropathy (DSPN) [19,38,39]. Hyperglycemia increased ROS production, accompanied by a decrease in mitochondrial membrane potential, in forskolin-treated IMS32 cells (Fig. 3), indicating that hyperglycemia enhances ROS production during the maturation of IMS32 cells.

**Fig. 3:**
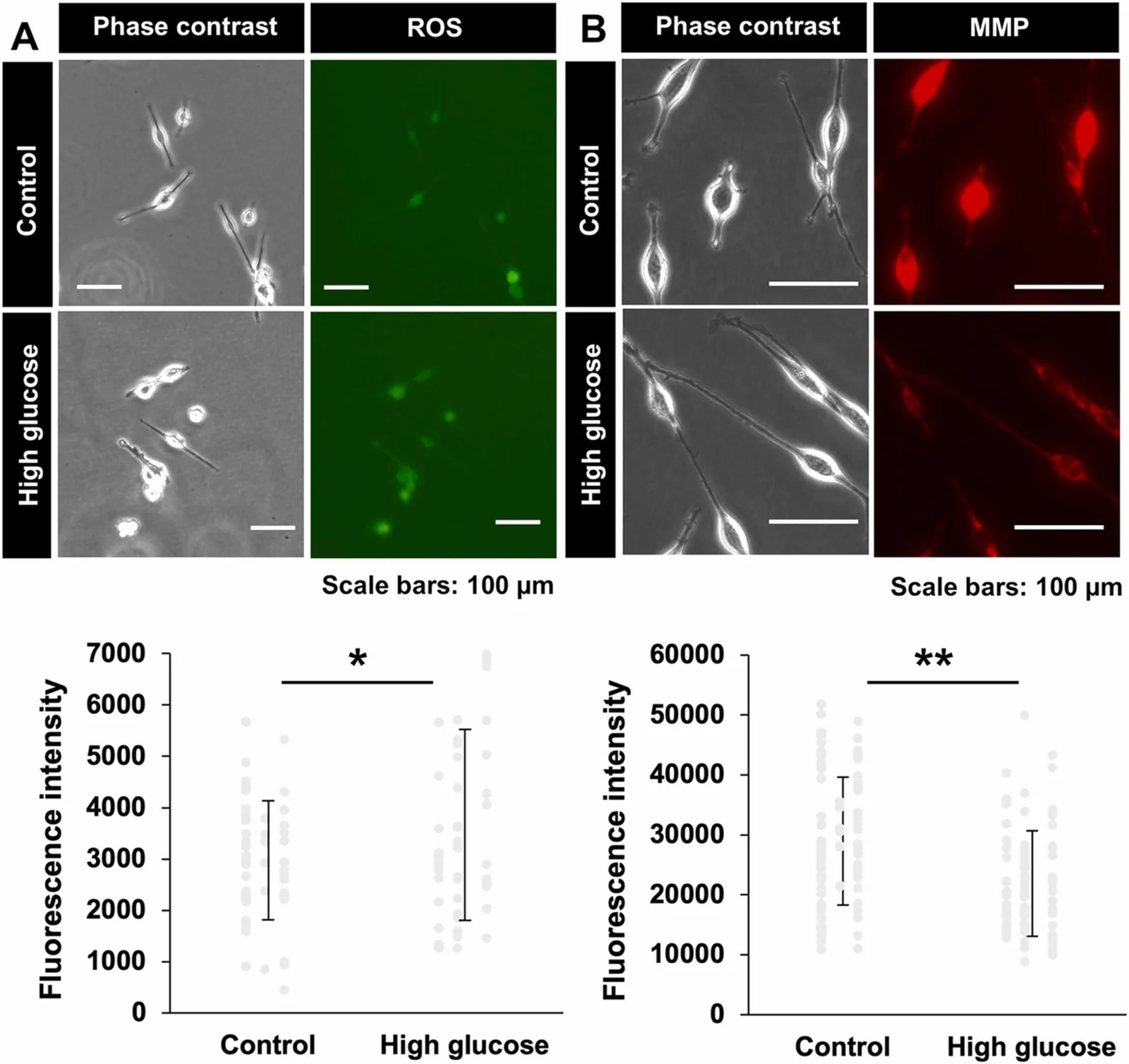
Hyperglycemia increased ROS production and impaired mitochondrial function in IMS32 cells treated with forskolin. Representative phase-contrast images and fluorescence images of ROS production (A) or mitochondrial membrane potential (MMP; B) in IMS32 cells exposed to normal or high-glucose conditions are shown. The graphs show the fluorescence intensity of ROS production (A) and MMP (B) under both conditions. Values represent the mean ± SD, with individual values depicted as circles (n = 50 (A) or 80 (B) cells). *p < 0.05, **: *p* < 0.01.

Nuclear factor-erythroid 2–related factor 2 (Nrf2) is a master regulator of the antioxidative stress response via binding to antioxidant response elements (AREs) [40,41]. Therefore, a promoter assay driven by AREs was conducted in IMS32 cells under hyperglycemic conditions. The proportion of EGFP-positive cells driven by AREs was significantly increased under hyperglycemic conditions (Fig. 4). The results suggest that the ARE promoter assay reflects ROS production and the antioxidative response, and suggest that oxidative stress suppresses the maturation process in IMS32 Schwann cells treated with forskolin under hyperglycemic conditions.

**Fig. 4:**
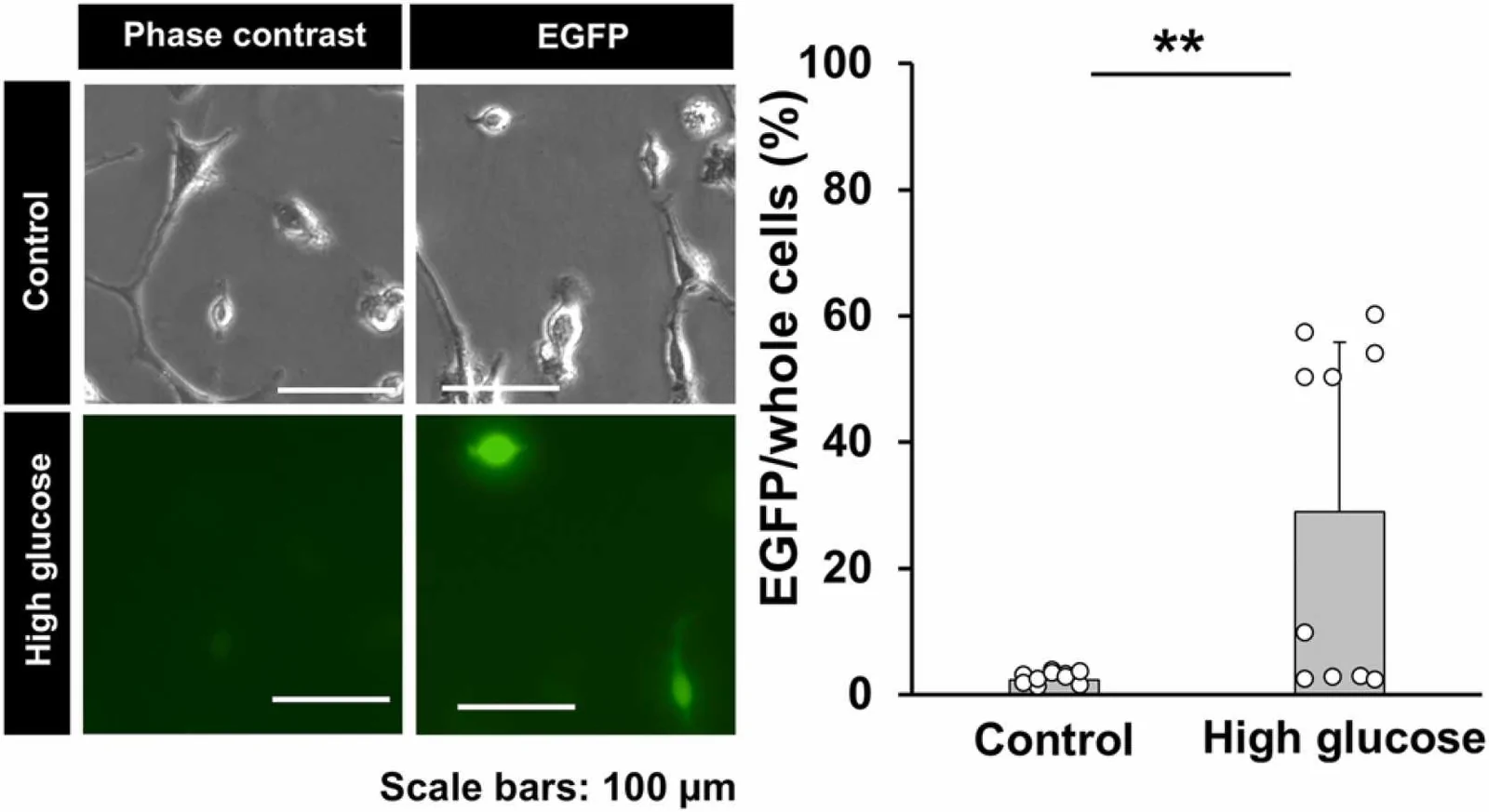
Hyperglycemia increased EGFP-positive cells driven by AREs in IMS32 cells treated with forskolin. Representative phase-contrast and EGFP images of IMS32 cells exposed to normal or high-glucose conditions are shown. EGFP-positive cells regulated by antioxidative response elements (AREs) are depicted. The bar graphs show the ratio of EGFP-positive cells under these conditions. Values represent the mean ± SD, with individual values depicted as circles (10 fields). **: *p* < 0.01.

### Pyruvate supplementation restored the impairment of IMS32 maturation under high-glucose conditions

Exogenous pyruvate is a potential candidate for DSPN treatment due to its antioxidative activity and ability to maintain glucose metabolism [37]. Therefore, we investigated whether pyruvate supplementation could ameliorate the impaired maturation of IMS32 cells under hyperglycemia. Pyruvate supplementation restored process length and maturation of IMS32 cells under high-glucose conditions (Figure S9). These results indicate that exogenous pyruvate ameliorates the impairment of the maturation process in IMS32 cells treated with forskolin.

To confirm this effect, promoter assays and immunoblotting were conducted in IMS32 cells supplemented with exogenous pyruvate. EGFP-expressing cells driven by the MBP, CNPase, or GFAP promoters were restored by pyruvate treatment (Fig. 5 and S10). Immunoblot analysis of MBP, CNPase, and GFAP also indicated prevention of the decreases observed under hyperglycemic conditions **(**Fig. 6). These data indicate that exogenous pyruvate may reverse hyperglycemia-induced deficits during maturation in IMS32 cells.

**Fig. 5:**
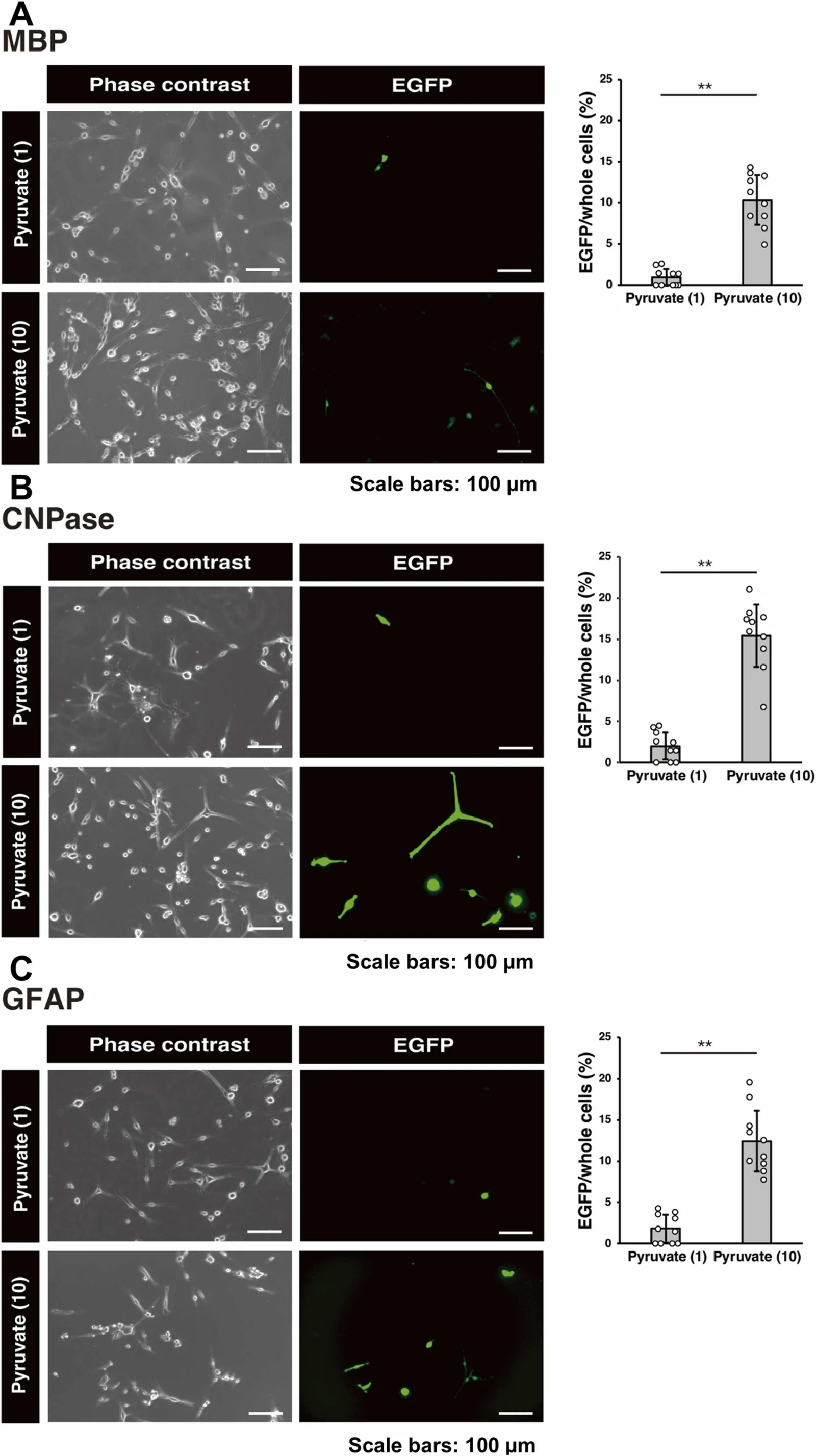
Exogenous pyruvate recovered EGFP-positive cells driven by MBP, CNPase, or GFAP promoters in forskolin-treated IMS32 cells. Representative phase-contrast and EGFP images of IMS32 cells exposed to high-glucose conditions containing 1 mM pyruvate [Pyruvate (1)] or 10 mM Pyruvate [Pyruvate (10)] are shown. EGFP-positive cells driven by MBP (A), CNPase (B), or GFAP (C) promoters are depicted. The bar graphs show the ratio of EGFP-positive cells under normal or high-glucose conditions. Values represent the mean ± SD, with individual values depicted as circles (n = 10 fields). ** *p* < 0.01.

**Fig. 6:**
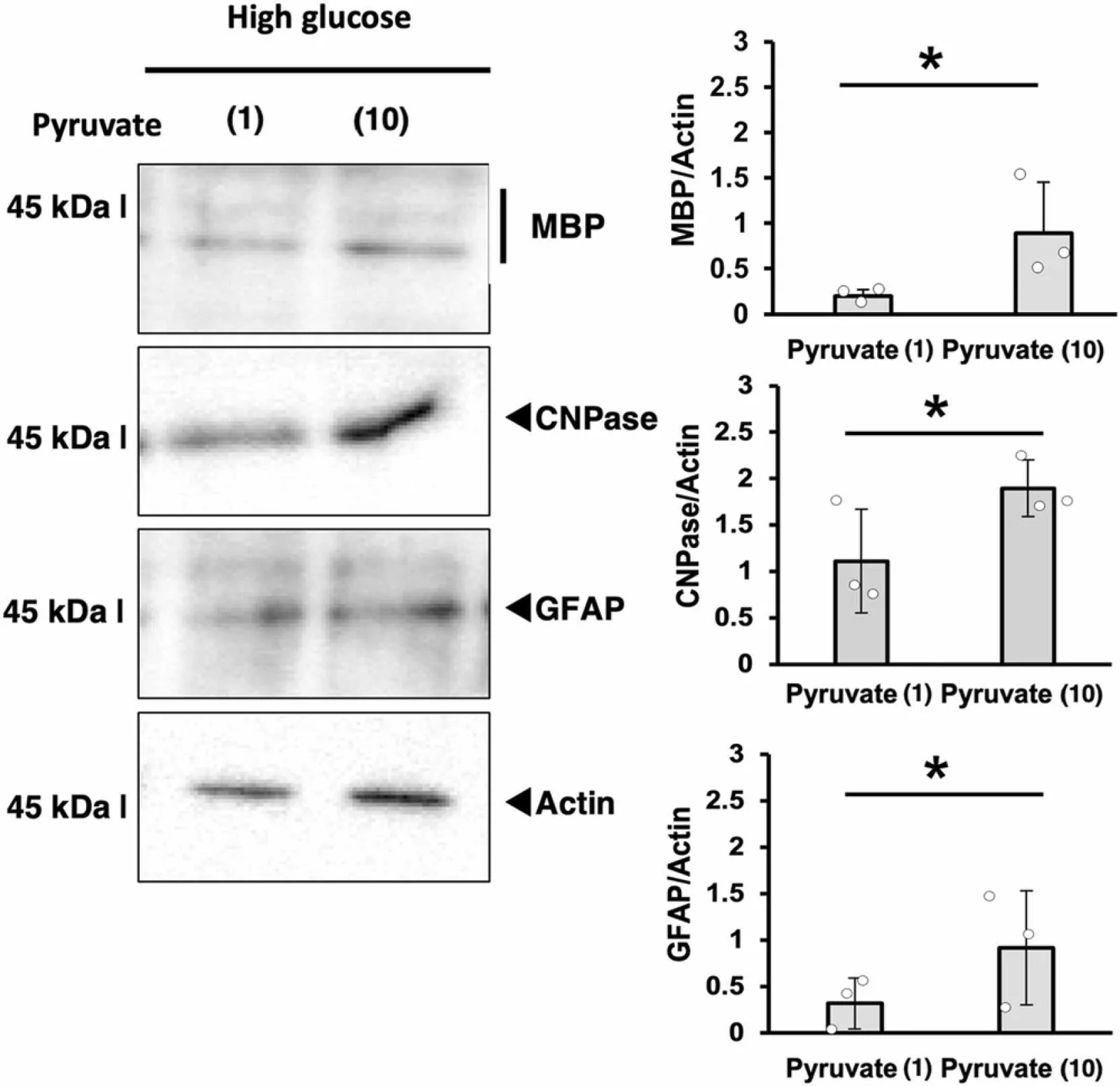
Pyruvate supplementation restored Schwann cell marker expression in forskolin-treated IMS32 cells under hyperglycemia. Representative bands of MBP, CNPase, GFAP, and Actin in IMS32 cells exposed to high-glucose conditions containing pyruvate at final concentration of 1 mM [Pyruvate (1)] or 10 mM [Pyruvate (10)] are shown. The bar graphs show the ratio of the intensity of each protein normalized by the intensity of Actin. Actual blots for MBP, CNPase, GFAP, and Actin are shown in Fig. S13. Values represent the mean ± SD, with individual values depicted as circles (n = 3). *p < 0.05.

### Pyruvate supplementation protected IMS32 cells against oxidative stress under hyperglycemic conditions

To examine the effect of exogenous pyruvate on oxidative stress, ARE-driven promoter assays were performed alongside measurements of ROS production and mitochondrial membrane potential. Pyruvate administration restored ROS levels and mitochondrial membrane potential (Fig. 7). The increase in EGFP-positive cells promoted by AREs was also reversed by pyruvate supplementation (Fig. 8). These results suggest that exogenous pyruvate mitigates oxidative stress and restores IMS32 cell maturation under hyperglycemic conditions. N-acetyl L-cysteine (NAC) is a well-established antioxidant. To further examine the involvement of oxidative stress, the effect of NAC on IMS32 cell maturation was assessed using promoter assays. NAC supplementation under hyperglycemia recovered a proportion of EGFP-positive cells driven by GFAP, CNPase and MBP promoters (Figure S11). These findings support the involvement of oxidative stress in hyperglycemia-impaired Schwann cell maturation.

**Fig. 7:**
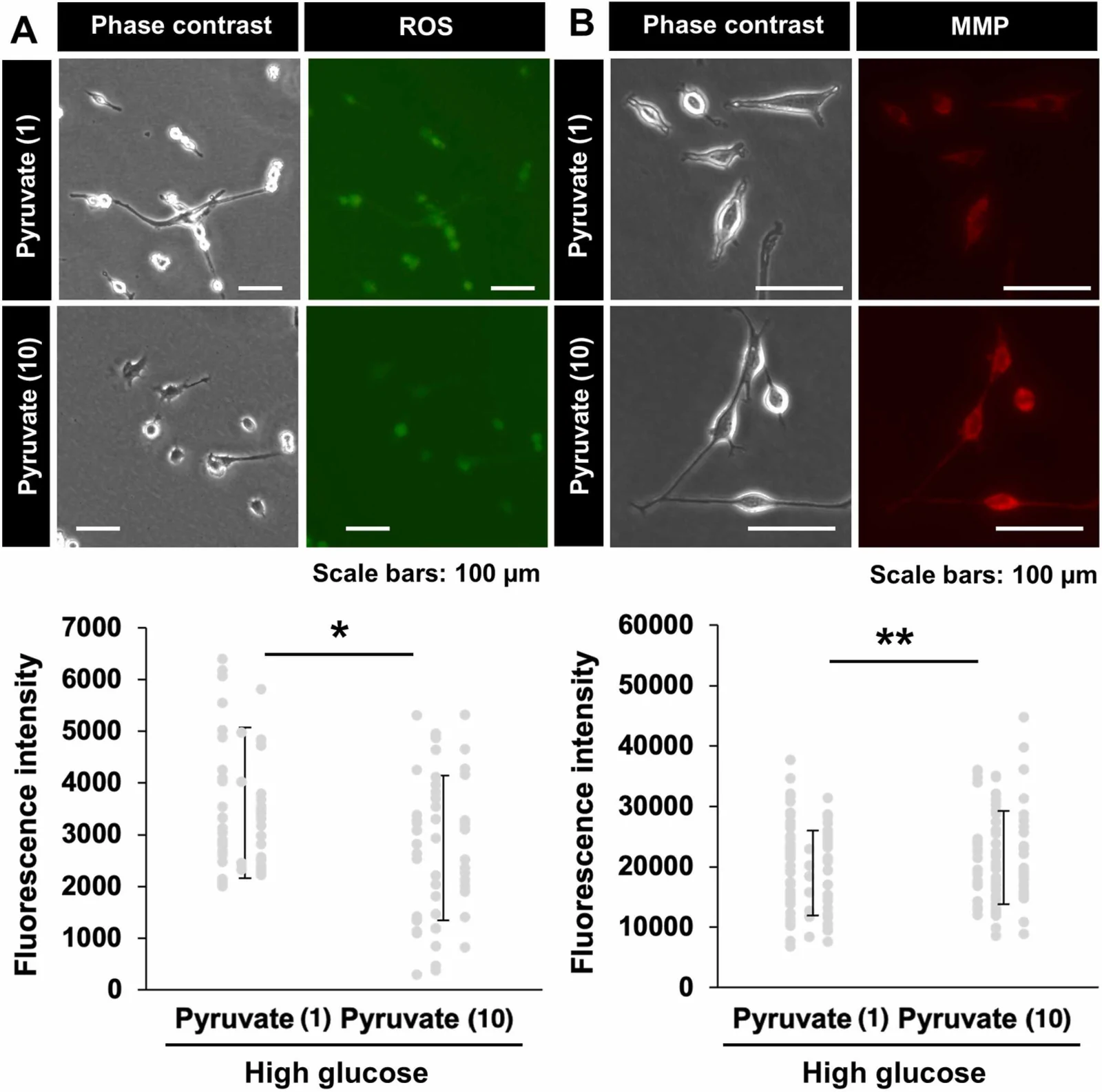
Exogenous pyruvate ameliorated hyperglycemia-induced ROS production and mitochondrial dysfunction in forskolin-treated IMS32 cells. Representative phase-contrast images and fluorescence images of ROS production (A) or mitochondrial membrane potential (MMP; B) in IMS32 cells exposed to high-glucose conditions containing pyruvate at final concentration of 1 mM [Pyruvate (1)] or 10 mM [Pyruvate (10)] are shown. The graphs show fluorescence intensity of ROS production (A) and MMP (B) under these conditions. Values represent the mean ± SD, with individual values depicted as circles (n = 50 (A) and 80 (B) cells). *p < 0.05, **: *p* < 0.01.

**Fig. 8:**
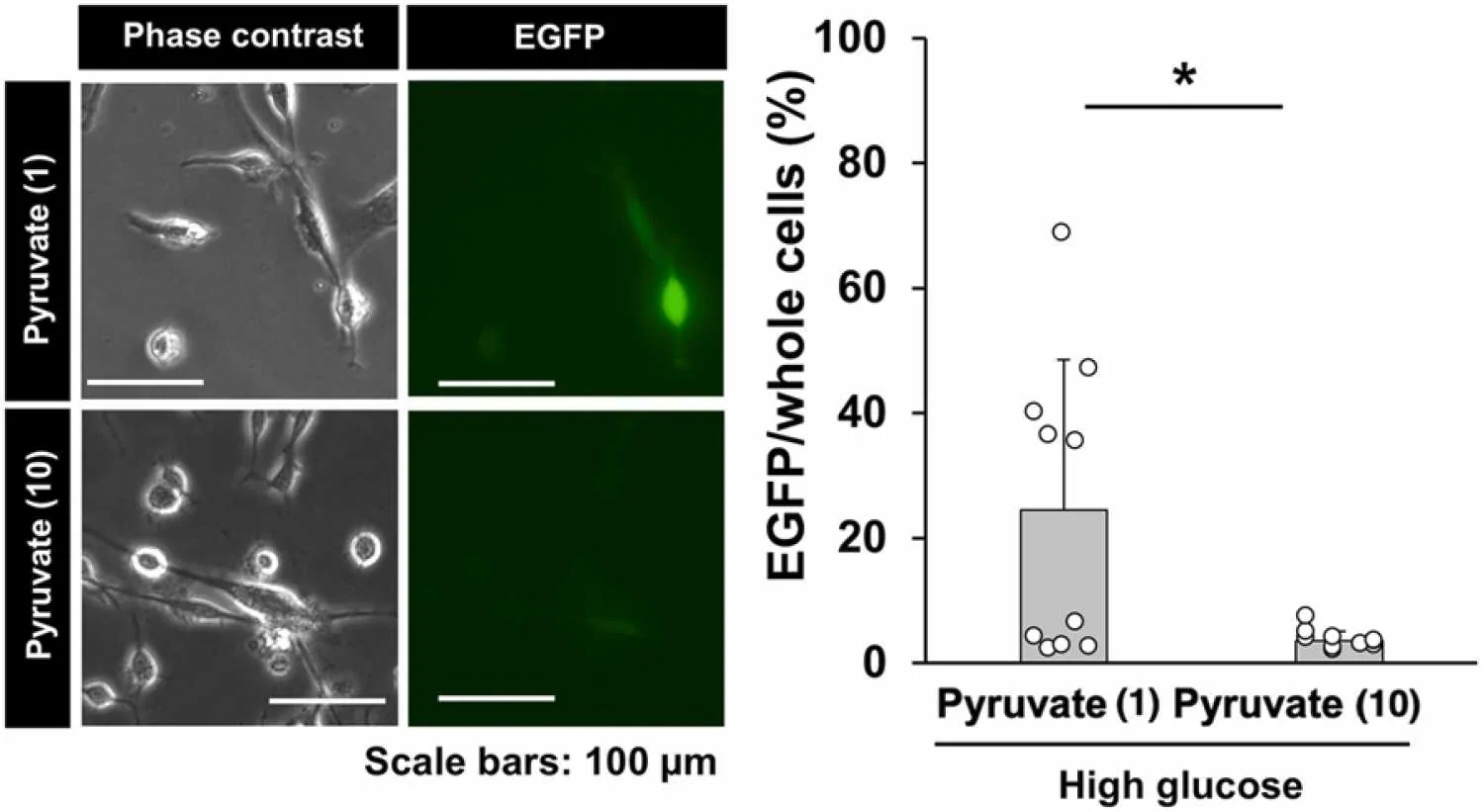
Exogenous pyruvate recovered EGFP-positive cells driven by AREs in forskolin-treated IMS32 cells under hyperglycemia. Representative phase-contrast and EGFP images of IMS32 cells exposed to high-glucose conditions containing pyruvate at final concentration of 1 mM [Pyruvate (1)] or 10 mM [Pyruvate (10)] are shown. EGFP-positive cells expressing under antioxidative response elements (AREs) are represented. The bar graphs show the ratio of EGFP-positive cells under these conditions. Values represent the mean ± SD, with individual values depicted as circles (n = 10 fields). * *p* < 0.05.

## Discussion

We examined the relationship between maturation and hyperglycemia in IMS32 Schwann cells, demonstrating that hyperglycemia impairs the maturation process in forskolin-treated IMS32 cells. We also showed that exogenous pyruvate may restore impaired maturation, at least in part, through its antioxidative activity. Our study suggests that hyperglycemia-induced impairment of Schwann cell maturation represents a novel pathogenic mechanism of DSPN.

In the present study, the term "Schwann cell maturation" refers to a sequential process including morphological differentiation, activation of maturation-associated genes, and expression of corresponding proteins, rather than a single biological endpoint. Process elongation was used as an early morphological indicator of Schwann cell maturation based on previously reported neuron-free differentiation methods [42,43]. In Schwann cells, morphological maturation is considered an early event, which precedes the sequential expression of maturation-associated genes and proteins, including GFAP, CNPase, and MBP. Thus, although approximately 80% of IMS32 cells exhibited process elongation after forskolin treatment, only a subset of these cells had progressed to a stage expressing maturation markers. The consistency between the promoter assay and immunoblotting findings further supports the validity of these complementary approaches for evaluating Schwann cell maturation.

IMS32 cells treated with forskolin upregulate Schwann cell markers and undergo morphological changes similar to primary cultured Schwann cells [44,45]. Hyperglycemia also reduced cell process length in forskolin-treated IMS32 cells. These results indicate that IMS32 cells retain an immature phenotype and that high-glucose conditions impair their maturation processes.

Excessive ROS production is a major pathogenic mechanism in DSPN and contributes to demyelination in the PNS [46]; however, the effect of hyperglycemia on Schwann cell maturation has remained unclear. In other cell types, such as osteoblasts, Nrf2 mitigates ROS production during differentiation [47]. Our ARE-driven promoter assays, which reflect Nrf2 activity, also showed a correlation between the expression and ROS production during Schwann cell maturation. These findings indicate that oxidative stress may impair the maturation process in several types of cells.

Endogenous pyruvate is the end product of glycolysis and is converted to lactate or acetyl-CoA for entry into the tricarboxylic acid cycle. Hyperglycemia has been reported to impair glycolytic flux through inhibiting GAPDH activity, leading to limiting pyruvate production in glycolysis [14,16,48]. In contrast, exogenous pyruvate is transported into cells through monocarboxylate transporters [49], thereby directly supplying intracellular pyruvate. Exogenous pyruvate also plays a crucial role in protecting cells against oxidative stress through both direct and indirect pathways. Pyruvate can function as an H_2_O_2_ scavenger [50], upregulate antioxidant or anaerobic glycolysis–related genes [51,52], and maintain flux through glycolysis, glycolytic collateral pathways, and the tricarboxylic acid cycle [14,16,37]. Pyruvate also has a differential role in regulating the antioxidative stress response with or without nuclear factor kappa-light-chain-enhancer of activated B cells translocation in fibroblasts and embryonic stem cells, respectively [51,53]. Ethyl pyruvate, a derivative of pyruvate, enhances Nrf2 translocation from the cytoplasm to the nucleus and its binding to AREs, resulting in the induction of antioxidative genes such as heme oxygenase-1 and glutathione S-transferase [52]. These findings indicate that pyruvate maintains cellular oxidative stress at cell-type-specific pathways through different mechanisms. The present findings using NAC support the idea that the protective effects of exogenous pyruvate on Schwann cell maturation are associated with attenuation of ROS.

In the present study, pyruvate administration decreased EGFP-positive cells driven by AREs, indicating that in IMS32 cells, pyruvate may function as a ROS scavenger or glucose metabolism regulator rather than by inducing ARE-mediated antioxidant gene expression. Together with our previous studies [14,16,37], these results suggest that exogenous pyruvate regulates ROS production in Schwann cells by reducing oxidative stress and maintaining glucose metabolism under hyperglycemic conditions.

Schwann cells support neuronal survival and function by providing metabolites, promoting axonal growth, and reducing oxidative stress [[54], [55], [56], [57]]. Proper maturation of Schwann cells is essential for them to provide these supportive functions [56,57]. Hyperglycemia impairs the maturation process and enhances dedifferentiation and abnormal myelination in Schwann cells. We suggest that these dysfunctions are the important contributors to DSPN, and protecting Schwann cells from hyperglycemia-induced stress may represent a pathogenesis-based therapeutic target.

The present study has several limitations. The impairment of forskolin-enhanced IMS32 Schwann cell maturation was observed at glucose concentration of 25 mM or higher, and pyruvate treatment at final concentration of 5 and 10 mM restored the maturation under hyperglycemia, at least partially, through its antioxidative activity. Although pyruvate administration into streptozotocin-induced diabetic mice improves neurite degeneration and sensory sensation [37], the beneficial effects of pyruvate in Schwann cell remain unclear. In addition, the glucose and pyruvate concentrations used in this study are supraphysiological. Therefore, further study using in vivo models is needed to determine whether the mechanisms identified in the present study reflect Schwann cell maturation in vivo under diabetic conditions.

DCFH-DA is widely used as an indicator of intracellular oxidative stress; however, it remains a non-specific probe and is susceptible to experimental artifacts. Although the NAC experiments support the involvement of oxidative stress in hyperglycemia-impaired Schwann cell maturation, further studies are required to fully elucidate the underlying mechanisms by which hyperglycemia-induced oxidative stress impairs Schwann cell maturation.

In addition, the distribution of EGFP-positive cells driven by ARE promoter appeared to be divided into groups with a lower or higher proportion. Although the basis for this distribution remains unclear, it is possible that differences in the maturation status of forskolin-treated IMS32 cells may contribute to this observation.

In summary, hyperglycemia impaired maturation and elevated oxidative stress in IMS32 Schwann cells. Exogenous pyruvate ameliorated these abnormalities under high-glucose conditions. These findings suggest that oxidative stress may contribute to impairment of Schwann cell maturation and to the pathogenesis in DSPN.

## CRediT authorship contribution statement

**Hideji Yako:** Writing – review & editing, Writing – original draft, Visualization, Supervision, Project administration, Methodology, Funding acquisition, Conceptualization. **Sana Hattori:** Visualization, Validation, Investigation, Formal analysis. **Sayaka Iwamoto:** Visualization, Validation, Investigation, Formal analysis. **Tatsuya Ooki:** Visualization, Validation, Investigation, Formal analysis. **Waraku Nagatani:** Validation, Investigation, Formal analysis. **Yuika Kaburaki:** Visualization, Validation, Investigation. **Yuki Miyamoto:** Writing – review & editing, Conceptualization. **Kazunori Sango:** Writing – review & editing, Conceptualization. **Junji Yamauchi:** Writing – review & editing, Supervision, Methodology, Funding acquisition, Conceptualization.

## Funding

This work was supported by Grants-in-Aid for Scientific Research from the Japanese Ministry of Education, Culture, Sports, Science and Technology (MEXT) and Grants-in-Aid for Medical Scientific Research from the Japanese Ministry of Health, Labor and Welfare (MHLW). This work was also supported by 10.13039/501100003382Core Research for Evolutional Science and Technology (CREST) of the 10.13039/501100002241Japan Science and Technology Agency (JST) as well as by Takeda Science Foundation, B, and C.

## Declaration of Competing Interest

The authors declare the following financial interests/personal relationships which may be considered as potential competing interests: Hideji Yako reports financial support was provided by Grants-in-Aid for Scientific Research from the Japanese Ministry of Education, Culture, Sports, Science and Technology (MEXT). Hideji Yako reports financial support was provided by Takeda Science Foundation. Junji Yamauchi reports financial support was provided by ng Sources List Add a Funding Source Grants-in-Aid for Scientific Research from the Japanese Ministry of Education, Culture, Sports, Science and Technology (MEXT). Junji Yamauchi reports financial support was provided by Grants-in-Aid for Medical Scientific Research from the Japanese Ministry of Health, Labor and Welfare (MHLW). Junji Yamauchi reports financial support was provided by Core Research for Evolutional Science and Technology (CREST) of the Japan Science and Technology Agency (JST). Junji Yamauchi reports financial support was provided by Takeda Science Foundation. If there are other authors, they declare that they have no known competing financial interests or personal relationships that could have appeared to influence the work reported in this paper.

## Data Availability

All data presented in this paper are available in the manuscript or from the corresponding author (H. Y.).
