# Supplementary material for: Exogenous pyruvate protects IMS32 Schwann cells from impairing the maturation process under hyperglycemia
Source: J Physiol Sci. 2026 Oct 3;76(3):100104. doi: 10.1016/j.jphyss.2026.100104 (PMC13672852; doi:10.1016/j.jphyss.2026.100104)
Supplement: Supplementary file 1 [file mmc1.docx]

**Exogenous pyruvate protects IMS32 Schwann cells from impairing the maturation process under hyperglycemia**

Hideji Yako^1, 2*^, Sana Hattori^1, 2^, Sayaka Iwamoto^1, 2^, Tatsuya Ooki^1, 2^, Waraku Nagatani^1, 2^, Yuika Kaburaki^1, 2^, Yuki Miyamoto^1, 2, 3^, Kazunori Sango^4^, and Junji Yamauchi^1, 2, 5*^

^1^Laboratory of Molecular Neuroscience and Neurology and ^2^Laboratory for Drug Target Discovery, Tokyo University of Pharmacy and Life Sciences, Horinouchi, Hachioji, Tokyo 192-0392, Japan; ^3^Department of Pharmacology, National Research Institute for Child Health and Development, Okura, Setagaya, Tokyo 157-8535, Japan; ^4^ Brain Metabolic Regulation Group Frontier Laboratory, Department of Psychiatry and Behavioral Sciences, Tokyo Metropolitan Institute of Medical Science, Tokyo 156-8506, Japan; ^5^Department of Biological Science, Tokyo College of Biotechnology, Kitakojiya, Oota, Tokyo 144-0032, Japan

**Figure S1. The proportion of EGFP-positive IMS32 cells.**

Representative phase contrast and EGFP images of IMS32 cells transfected with [Vector (+)] or without [Vector (-)] EGFP-C1 plasmid. The bar graphs exhibit the ratio of EGFP-positive cells 0, 1 or 2 days after transfection. Values represent the mean ± SD, with individual values depicted as circles (n = 10 fields).

**Figure S2. The assessment of transfection efficiency and cell viability in IMS32 cells under experimental conditions**

Transfection efficiency (**A**) and cell viability of IMS32 cells at 2 days after treatment with forskolin under 5.6, 25, 50 or 100 mM glucose conditions supplemented with 1, 5 or 10 mM pyruvate, 100 mM mannitol or 150, 300 or 600 μg/ml N-acetyl L-cysteine (NAC) at final concentration are shown. Values represent the mean ± SD, with individual values depicted as circles (n = 10 fields (**A**) and 4 wells (**B**)).

**Figure S3. The morphological changes of forskolin-treated IMS32 cells during maturation process**

Representative phase contrast images (**A**) of IMS32 cells at 0, 1, 5, or 24 hours after treatment with forskolin. The bar graphs exhibit the ratio of the mature IMS32 cells and the process length in the absence (**B**) or presence (**C**) of forskolin. For single-cell analysis, the distribution of process length was exhibited at each time (**D**). The same measurements were used to calculate the mean and single cell process length (**C** and **D**). Values represent the mean ± SD, with individual values depicted as circles (n = 10 fields and 50-81 cells). **p* < 0.05, **: *p* < 0.01

**Figure S4. The morphological changes of IMS32 cells under hyperglycemia**

Representative phase contrast images of IMS32 cells treatment with forskolin 0, 1, or 2 days after exposure to normal or high- glucose conditions (**A**). The bar graphs exhibit the ratio of the mature IMS32 cells (**B**) and the process length (**C**) under these conditions. Values represent the mean ± SD, with individual values depicted as circles (n = 10 fields). **p* < 0.05, **: *p* < 0.01

**Figure S5. The proportion of the number of EGFP-positive cells derived from MBP promoter.**

Representative phase contrast and EGFP images of IMS32 cells at 0, 1, or 2 days after transfection of plasmid deriving EGFP under MBP promotor in the absence or presence with forskolin. The bar graphs exhibit the ratio of EGFP-positive cells in the presence or absence of forskolin. Values represent the mean ± SD, with individual values depicted as circles (n = 10 fields). **p* < 0.05, **: *p* < 0.01

**Figure S6. The proportion of the number of EGFP-positive cells derived from MBP, CNPase or GFAP promoter at various glucose concentrations.**

Representative phase contrast and EGFP images of IMS32 cells after transfection of plasmid deriving EGFP under MBP, CNPase or GFAP promotors under 5.6, 25, 50 or 100 mM glucose. The bar graphs exhibit the ratio of EGFP-positive cells under each condition. Values represent the mean ± SD, with individual values depicted as circles (n = 10 fields). **: *p* < 0.01

**Figure S7. Mannitol-induced osmotic stress had no effect on Schwann cell maturation.**

Representative phase contrast and EGFP images of IMS32 cells exposed to normal glucose, high-glucose or mannitol conditions are shown. EGFP-positive cells driven by GFAP promoter are represent. The bar graphs exhibit the ratio of EGFP-positive cells under these conditions. Values represent the mean ± SD, with individual values depicted as circles (n = 10 fields). **p* < 0.05, **: *p* < 0.01

**Figure S8. Mannitol-induced osmotic stress had no effect on Schwann cell maturation.**

Representative phase contrast and EGFP images of IMS32 cells exposed to normal glucose, high-glucose or mannitol conditions are shown. EGFP-positive cells driven by MBP promoter are represent. The bar graphs exhibit the ratio of EGFP-positive cells under these conditions. Values represent the mean ± SD, with individual values depicted as circles (n = 10 fields). **p* < 0.05, **: *p* < 0.01

**Figure S9. The morphological changes of pyruvate-supplemented IMS32 cells under hyperglycemia**

Representative phase contrast images of IMS32 cells supplemented with pyruvate 0, 1, or 2 days after exposure to high-glucose conditions (**A**). The bar graphs exhibit the ratio of the mature IMS32 cells (**B**) and the process length (**C**) under high-glucose conditions supplemented without [Pyruvate (1)] or with [Pyruvate (10)] pyruvate at 0, 1, or 2 days. Values represent the mean ± SD, with individual values depicted as circles (n = 10 fields). **p* < 0.05, **: *p* < 0.01

**Figure S10. The proportion of the number of EGFP-positive cells derived from MBP, CNPase or GFAP promoter under high-glucose conditions in the presence of various pyruvate concentration.**

Representative phase contrast and EGFP images of IMS32 cells after transfection of plasmid deriving EGFP under MBP, CNPase or GFAP promotors under high-glucose conditions in the presence of 1, 5 or 10 mM pyruvate. The bar graphs exhibit the ratio of EGFP-positive cells under each condition. Values represent the mean ± SD, with individual values depicted as circles (n = 10 fields). **: *p* < 0.01

**Figure S11. The proportion of the number of EGFP-positive cells derived from MBP, CNPase or GFAP promoter under high-glucose conditions in the presence of various N-acetyl L-cysteine concentration.**

Representative phase contrast and EGFP images of IMS32 cells after transfection of plasmid deriving EGFP under MBP, CNPase or GFAP promotors under high-glucose conditions in the presence of 0, 150, 300 or 600 μg/ml N-acetyl L-cysteine**.** The bar graphs exhibit the ratio of EGFP-positive cells under each condition. Values represent the mean ± SD, with individual values depicted as circles (n = 10 fields). **: *p* < 0.01

**Figure S12. The actual immunoblotting images of MBP, CNPase, GFAP, and Actin.**

Full length of membranes of MBP, CNPase, GFAP, and Actin presented in **Figure 2** are shown. The black boxes indicate the areas represented in **Figure 2**.

**Figure S13. The actual immunoblotting images of MBP, CNPase, GFAP, and Actin.**

Full length of membranes of MBP, CNPase, GFAP, and Actin presented in **Figure 6** are shown. The black boxes indicate the areas represented in **Figure 6**.
